# The long non coding RNA H19 as a biomarker for breast cancer diagnosis in Lebanese women

**DOI:** 10.1038/s41598-020-79285-z

**Published:** 2020-12-17

**Authors:** Tamina Elias-Rizk, Joelle El Hajj, Evelyne Segal-Bendirdjian, George Hilal

**Affiliations:** 1 School of Medicine, Lebanese American University, Beirut, Lebanon; 2 Natural Sciences Department, Lebanese American University, Beirut, Lebanon; 3 Cancer and Metabolism Laboratory, Faculty of Medicine, Saint-Joseph University, Mar Mikhaël, Beirut, Lebanon; 4 Team: Cellular Homeostasis, Cancer, and Therapies, INSERM UMR-S 1124, Université de Paris, Paris, France; 5 Université de Paris, Paris Sorbonne Cité, Paris, France; 6 BioMedTech Facilities, CNRS UMS2009/INSERM US36, Université de Paris, Paris, France

**Keywords:** Breast cancer, Cancer, Biomarkers, Medical research, Oncology, Risk factors

## Abstract

Breast cancer is the most common cancer in women worldwide. Minimally invasive percutaneous image-guided biopsies are the current cornerstone in the diagnosis of breast lesions detected on mammography/ultrasonography/MRI or palpable clinically. However, apparently benign breast disease seen on benign biopsies is a limiting factor for diagnosis and a risk factor of breast cancer especially in the high-risk category patients. Hypothesizing that molecular changes often occur before morphological variations, the levels of the LncRNA H19 were measured in anonymous tissues obtained from 79 women’s image guided breast biopsies, and correlated with cancer progression and aggressiveness. Using a double-blinded approach, H19 might be attributed an interesting role of a more sensitive biomarker in core breast biopsies, independently of the radiological/clinical classification and distant from the clinical management. We established different thresholds for H19 levels in normal versus proliferative, versus malignant tissues. Additionnally, H19 could act as an intra-group risk marker categorizing the biopsies in normal versus benign, versus precancerous breast tissue, and as a prognostic factor in cancerous lesions discriminating aggressive versus nonaggressive lesions. Our study suggests that the lncRNA H19 could be a potential marker for breast cancer diagnosis, prognosis and risk management.

## Introduction

Breast cancer is the most popular cancer type in women worldwide^1,2^. Its frequency has increased in both developed and developing countries, primarily because of advances in diagnostic methods. Due to the detection of early-stage tumors by breast cancer screening, breast cancer mortality was significantly reduced as well as locoregional and distant recurrences prevalence^3^. Interval cancers occur in the age-specific screening group during the interval between two consequent screenings. They are characterized by very aggressive tumors with rapid grow^4^. They have worse prognostic factors, and their incidence could represent a good indicator of screening effectiveness^4^.

Mammography remains the gold standard of breast cancer detection, screening and diagnosis. Most patients with newly diagnosed breast cancer undergo imaging by mammography, ultrasonography or both. MQSA’s National Statistics stated the highly sensitivity (79%) and specificity (90%) of mammography, however lower in younger women and women with dense breast tissue^4^.

Breast Imaging Reporting and Database System (BI-RADS) is the standardised technique to describe and report findings on imaging^4^. BI-RADS 4 or highly suspicious BI-RADS category 5 lesions are correlated to histological analysis due to their important association with malignancy by 33–50% and 90% respectively^5,6^.

Percutaneous image guided biopsies are the cornerstone of histological diagnosis^6^. They replace surgical diagnostic biopsies for the majority of breast lesions^7^. To be able to characterize the lesion histologically and to plan overall oncological management for very large cancers, needle biopsy is performed. It allows the identification of the histological type and grade, basal subtype, hormonal and HER2 receptor status, as well as genetic profiling^5^.

In addition to possible technical limitations/errors in sampling, the presence of benign breast disease causes an inaccuracy in providing full characterization of the lesion by the biopsy^5,8,9^. It can underestimate the existence of disease such as the cases of complex sclerosing lesions, atypical ductal hyperplasia, papillary lesions, lobular carcinoma in situ (LCIS), radial scars, and phylloides tumors^5,10^. Breast cancer being a multifactorial disease, benign breast disease (BBD) is one of the most important risk factors for this malignancy^11^. It is mandatory to review the pathology results and correlate them with the clinical and radiological findings. This will avoid missing lesions or underestimating pathologies, particularly in the context of BBD, often present on benign breast biopsies^4,7,11^. Despite the frequency and high-risk nature of atypia in BBD, its biology is still poorly understood^8^. Better understanding of the natural history of atypical hyperplasia, ductal, and lobular^8,12^ and of the variations in the intrinsic biology of the tumors^13^ will advance both our understanding of breast carcinogenesis and our clinical management of high-risk patients^8,14^.

Taking into consideration the social and economical impact of breast cancer, it is mandatory to ameliorate the diagnosis, treatment, and prognosis of the patients^15^. It seems urgent to find a biomarker with high specificity and sensitivity to increase breast cancer early detection. Therefore, this will avoid advanced stages and worse prognosis^16^.

In molecular biology research, advents in whole genome and transcriptome sequencing techniques have brought long noncoding RNAs (lncRNAs) into the spotlight. Their critical role in normal development as well as the tumorigenesis process is being elucidated^17^. LncRNAs are emerging key players as biomarkers in breast cancer due to their key roles in a wide spectrum of cellular and developmental processes^18,19^. H19 was among the first reported long noncoding RNAs. It is highly expressed during embryological development and absent or greatly reduced in most adult tissues^20^. However, H19 is overexpressed in 73% of breast cancer tissues in comparison with healthy ones^21,22^. Its implication in tumorigenesis has been reported in many solid tumors such as bladder, prostate and breast cancers^21,23^. Many studies have shown an association between specific H19′s Single Nucleotide Polymorphisms (SNPs) and the overall cancer risk of squamous cell carcinoma, hepatocellular carcinoma, osteosarcoma, bladder cancer, gastric cancer, and breast cancer^24–29^.

Our results showed that the long noncoding RNA H19 measured in fresh breast biopsies could not only be a potential marker for cancer diagnosis, but also a good marker for subcatecorizing the lesions. Since the molecular changes occur way before the morphological changes in cancer lesions, the lncRNA H19 could also provide information on patient prognosis and possibly on patient follow up and treatment.

## Results

### H19 levels correlate with malignancy

**Figure 1:**
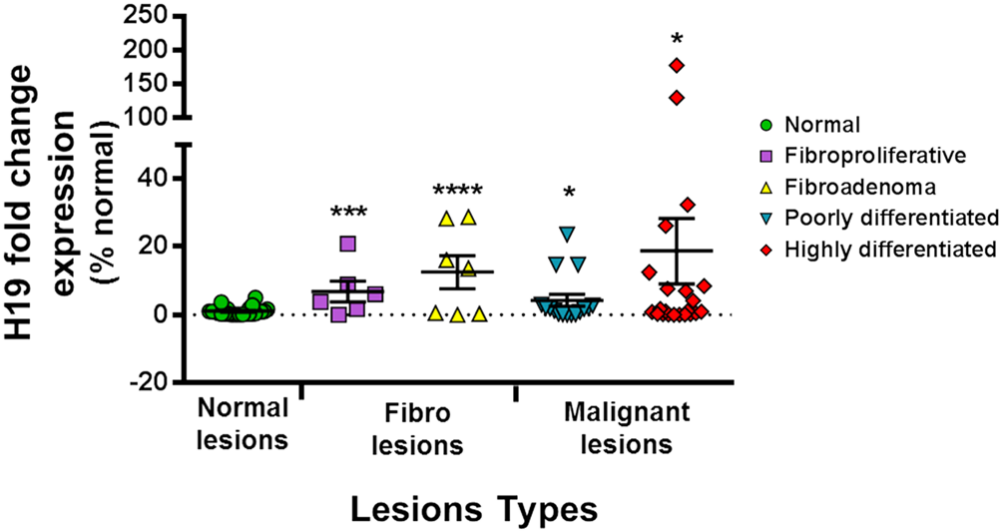
H19 levels are higher with malignancy degree. On a total of 79 biopsies, H19 fold change expressions were progressively higher in fibroproliferative lesions (6.9 ± 3.07), fibroadenoma lesions (12.5 ± 4.8) poorly differentiated lesions (4.3 ± 1.73) and highly differentiated lesions (18.8 ± 9.6) compared to normal lesions. Expression levels were normalized to GAPDH expression. Results were expressed as means ± SEM. ANOVA and t-test, *p < 0.05, ***p < 0.001, ****p < 0.0001.

H19 expression showed significant differences between H19 fold change levels amongst lesions in comparison with normal lesions. On a total of 34 samples showing higher H19 expression in comparison with the control group, 11 samples were among the fibro lesions group and 23 samples were among the malignant lesions group. Fibroproliferative lesions showed an H19 fold change level of 6.9 (mean ± 3.07), followed by the fibroadenoma lesions with a higher H19 fold change level of 12.5 (mean ± 4.8). These elevated values are of interest to be discussed. Interrestingly, H19 fold change levels in poorly differentiated lesions is 4.3 (mean ± 1.73) to a fold change mean of 18.8 (mean ± 9.6) in the highly differentiated malignant lesions (Fig. 1).

### H19 is considered as a risk factor for cancer development

**Figure 2:**
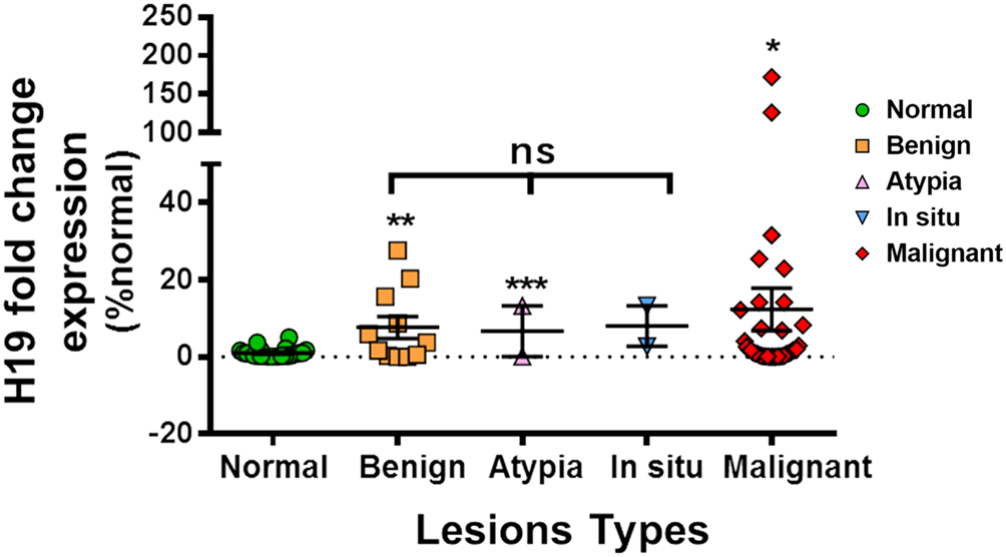
Three different intervals of H19 expression accordingly with three classes of cancer degeneration. H19 fold change expression levels in benign biopsies were the lowest (7.6 ± 2.85) followed by atypia and in situ lesions H19 fold change levels, while malignant lesions showed the highest H19 fold change expression (12.3 ± 5.5). Expression levels were normalized to GAPDH expression. Results were expressed as means ± SEM. ANOVA and t-test, *p < 0.05, **p < 0.01, ns p > 0.5.

As previously mentioned, our main challenge was to identify H19 levels thresholds, possibly existing in breast lesions’ intra-groups. On a total of 34 samples showing higher H19 expression in comparison with the control group, 8 lesions were among the benign group, 1 lesion was among the atypia group, 2 lesions were among the in situ group, and 23 lesions among the highly malignant one. We established two different intervals of H19 levels in the studied biopsies. All lesions with an H19 fold change level lower than 7.6 (± 2.85) corresponded to non-cancerous lesions. However, due to the limited number of lesions caracterised in pathology as atypia and in situ and have a possibility of cancer degeneration, the established fold change means need to be confirmed with a higher number of specimens. Moreover, malignant lesions showed the highest H19 fold change expression of 12.3 (± 5.5) (Fig. 2).

### H19 is high in fibrocystic lesions

**Figure 3:**
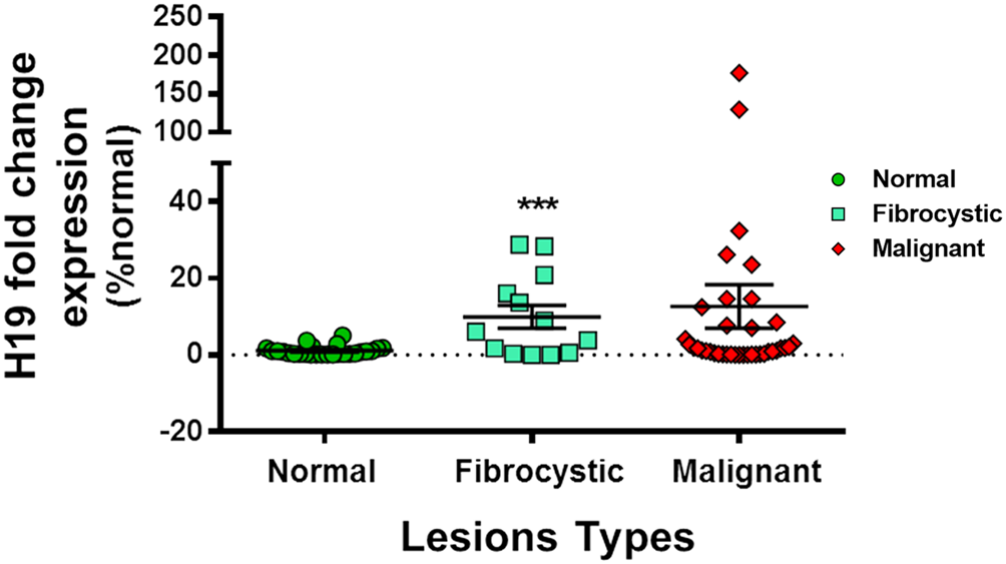
H19 levels are high in fibrocystic lesions. Fibrocystic lesions presented an H19 fold change expression level of 9.9 (9.9 ± 2.9) while malignant lesions had an H19 expression level of 12.34 (12.3 ± 5.5). Expression levels were normalized to GAPDH expression. Results were expressed as means ± SEM. ANOVA and t-test, ***p < 0.001.

We wanted to distinguish between the studied biopsies and check for any relevance of H19 expression levels in fibrocystic lesions compared to malignant ones. On a total of 34 samples showing higher H19 expression in comparison with the control group, 10 samples were among the fibrocystic group and 24 samples were among the malignant ones. We noticed relatively high levels of H19 expressions in fibrocystic lesions compared to normal lesions (9.9 ± 2.9). However, as previously mentioned, malignant lesions presented higher levels of H19 (12.3 ± 5.5) (Fig. 3). These results suggest that molecular markers and in our case H19 could be used as add on tool complimentary to the anapathology that gives benignity or malignancy diagnosis in order to distinguish amongst benign tissues the category having high levels of H19 and possibly indicating high proliferative cellular phenotype.

### H19 levels are correlated with differenciation and hormonal profile

**Figure 4:**
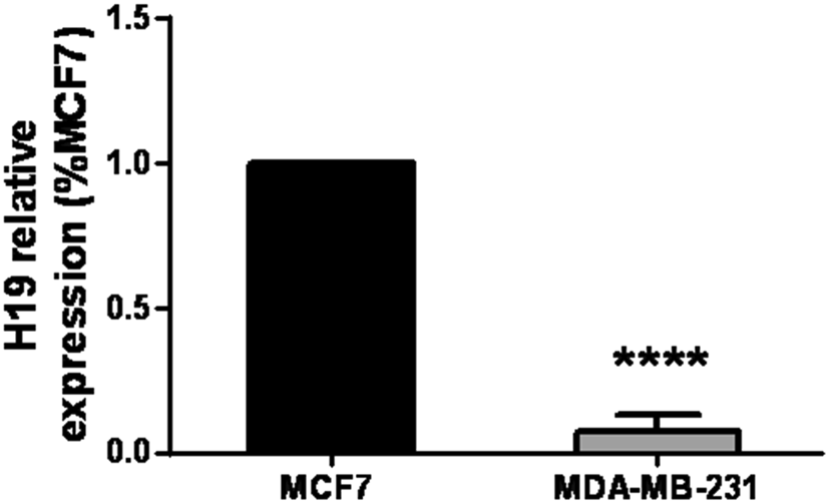
H19 basal levels in MCF-7 and MDA-MB-231 cells. MCF-7 triple positive cells present higher H19 basal levels in comparison with the MDA-MB-231 les differentiated and more invasive triple negative cells. Results were expressed as means ± SD. t-test, ****p < 0.0001**.**

To compare our findings with H19 basal levels in different established breast cancer cellular lines, we used the MCF-7 and MDA-MB-231 breast cancer cells present in our research unit. Triple positive, more differentiated MCF-7 cell line presented higher basal levels of H19 compared to the triple negative, less differentiated and more invasive MDA-MB-231 breast cancer cell line (Fig. 4).

## Discussion

Despite considerable advances in the development of therapies, improvements in the survival and morbidity/mortality prognosis of breast cancer patients have not followed^30,31^. A better understanding of the molecular biology of breast cancer added to a better characterization of the known and newly discovered potential markers, would be of importance for the care and treatment of breast cancer. This will improve the patient’s prognosis as well.

Long nocoding RNAs (lncRNAs) have been reported to serve as diagnostic and prognostic biomarkers of cancers^32^. They play vital roles in tumorigenesis and tumor progression. Our study focused on defining a possible role for the long noncoding RNA H19 as a potential prognostic biomarker in biopsied malignant breast tissue and as risk factor in non-cancerous biopsied lesions. Our hypothesis is based on the fact that molecular changes occur before morphologic and phenotype variations. When the histopathological analysis is unconclusive about the lesion’s type, H19 levels will be an added value to characterize the lesion and dictate the management in correlation with clinical/radiological suspicion. In these cases, H19 levels can contribute in the management of these patients. A close follow up can be decided in a low-risk patient. However, a further correlation with surgical resection in a high-risk patient would be recommended. Interrestingly, several studies have been performed to explore the diagnostic value of lncRNA H19 in cancer detection and diagnosis: high H19 serum levels in patients with certain myeloma and nonsmall cell lung carcinoma have been suggested to be useful for diagnosis and prognosis^33,34^. Thus, the long noncoding RNA H19 might be a candidate for the development of promising diagnostic modalities for several cancers^35^. The important network constructed between H19 and tumor suppressor genes, as well as oncogenes provides clues for crucial role of H19 in tumorigenesis^36^.

On a total of 79 biopsies, the assessment of H19 levels in tissues by qRT-PCR showed significant differences between the attributed H19 level means amongst lesions: in comparison to normal lesions (where the predominant tissue corresponds to fibrofatty breast tissue on histology), H19 fold change levels increased gradually in fibroproliferative lesions (fibrocystic/dystrophic changes as per the histology report) (6.9 ± 3.07), followed by fibroadenoma lesions (12.5 ± 4.8). These elevated values in biopsies classified histologically as not cancerous could be due to the hyper proliferative state of the cells. Fibroadenomas are lesions belonging to the panel of fibrodystrophic changes in benign breast pathology. Our study showed elevated levels of H19 in 11 benign fibrocystic lesions. Histology results of these lesions revealed the presence of inflammation. A possible correlation can be suggested: inflammation is being a risk factor of cancer degeneration. However, complex fibroadenomas can be associated with histological risk factors for breast cancer without being an independent risk factor^37^.

In case of a proven malignancy by histopathology, H19 will act as a prognostic factor. Low levels of H19 in cancerous biopsied lesions would be indicative of more aggressiveness. Less aggressiveness would be related to cancers showing high H19 expression. H19 levels would then dictate the therapeutical management. In patients with proven cancer and expressing low levels of H19, a radical surgical treatment would be an option to be considered. However, conservative surgical treatment would be more easily adopted in multidisciplinary discussions in patients having a cancer with high levels of H19. Interrestingly, poorly differentiated lesions had a lower H19 fold change level (4.3 ± 1.73) compared to highly differentiated ones (18.8 ± 9.6). Several studies have been used to explain the difference in H19 levels detected in biopsies, while first focusing on the correlation between H19 expression and the differentiated state of cancer cells^38,39^. Moreover, the expression of H19 has been correlated with hormone receptors^40^. This was confirmed in our breast cancer cellular lines, where triple positive and more differentiated MCF-7 cell line presented higher basal levels of H19 compared to the triple negative less differentiated MDA-MB-231 breast cancer cell line. Additionnally, overexpression of H19 in breast cancer has been significantly correlated with the presence of estrogen and progesterone receptors^21^. MCF-7/AdrVp breast cancer cells multi-resistant to several treatments showed a more abundant expression of H19 compared to parental MCF-7 cells^41^. Moreover, the degree of differentiation correlated to Ki 67^42^, hormone receptors, but also cell types, can explain, while overlapping, the different levels of H19′s expression. H19 has been reported to be associated with differentiation of luminal progenitor cells from estrogen-regulated cells^11,43^. For high-grade cancers in our study, we should try to explain the low levels of H19 by considering other factors. It is unusual for a simple mechanism to explain all of the changes in H19 expression levels that take place in the differentiated, aging, or neoplastic mammary gland^21^. H19’s basal expression level increases in normal breast tissue during adulthood. In addition, the loss of regulation of this gene in carcinomas seems to be the result of a puzzling assembly process. It reflects the fundamental relationships between cells of different phenotypes.

Our main challenge was to identify H19 levels thresholds, possibly existing in the different breast lesions’ intra-groups. We showed that all lesions with an H19 fold change level lower than 7.6 (± 2.85) corresponded to non-cancerous lesions. Moreover, malignant lesions showed the highest H19 fold change expression of 12.3 (± 5.5). However, due to the limited number of lesions characterized in anapathology as atypia and in situ with a possibility of cancer degeneration, the established fold change means need to be completed with a higher number of specimens. Also, the heterogeneity of the manipulated tissue constituted another limitation: the biopsy core is definetly obtained from the lesion itself and controlled by imaging, yet the many biopsy fragments target different areas in the same lesion. Knowing that only a small fragment is sent for molecular assessment, this could possibly create a discrepancy due to different histological phenotypes amongst the same lesion. Furthermore, Peng et al. have demonstrated that high levels of H19 in breast tumors can be indicators of poorer survival in 20 patients with breast cancer^31^. Shima et al. have also demonstrated this for 180 patients with breast cancer, correlating H19′s expression with poor survival. Survival was notably shorter in patients with triple negative breast cancer. Triple-negative breast cancer had the poorest prognosis because of its resistance to chemotherapy^30,44^. This confirms our results of H19 as prognostic in intra cancer group with different levels of expression in the different tumor categories.

Moreover, Wang et al. showed that percutaneous biopsies had good sensitivity and specificity with a cumulative sensitivity of 87% and a cumulative specificity of 98%. As mentionned, our study is not about questioning the role of pathology in the diagnosis of cancer. However, the majority (approximately 80%) of the histological results come out as benign^4^, and a biopsy not presenting a malignancy to the anatomopathology can consist of different kinds of “benign” tissues: fatty tissue, fibroglandular tissue, fibrocystic lesions, adenosis, papillomas, hyperplasias. In these different cases, the addition of a sensitive biomarker would affect the management of later-on follow up particularly in high-risk patients. It may increase our ability to detect breast cancer at an early stage in proliferative tissue that has been identified as benign. This will allow us to better assess risk factor in patients with dense breasts taking into account the predisposition to develop breast cancer^45,46^. Even more, long noncoding RNAs can serve as diagnostic and prognostic biomarkers in human cancers due to the fact that lncRNAs could be collected from body fluids like plasma and urine^16^. These markers could be found more sensitive than histopathology, particularly in the presence of benign breast disease highlighting potential risk factors for cancer or cancer precursors^47^. In addition, they can help to identify an aggressive disease or predict a metastasis. Zhang et al. assessed the possibility that H19 in plasma could serve as a biomarker for the diagnosis and monitoring of breast cancer^48^. H19 levels were significantly increased in the plasma of breast cancer patients compared to healthy volunteers. They could have a major impact in the management of the disease and its aftermath^36^. Yet, various applications (tumor profiling, risk of relapse or recurrence, detection of cancer at early stages) are possible, but many aspects still need to be explored before transferring these finding to clinical application^49^.

The results of this study are promising for a possible improvement in the management of breast cancer pathology. We identified the long noncoding RNA H19 as a potential marker for breast cancer in Lebanese women. H19 would have a role as a risk factor to manage high-risk women particularly in atypical or conflictual cases. It would be a prognosis factor in confirmed breast cancer to identify an aggressive disease or to predict a metastasis/recurrence. H19 could be granted more implication in the treatment if constituting a potential therapeutic target. However, in order to establish a better profile of H19, more molecular evaluation should be combined with clinical evaluation to distinguish its role in different breast tissues.

## Methods

### Study population

The study was approved by the Institutional Review Board (IRB) of the Lebanese American University (LAU) and the ethical committee of Saint-Joseph University (USJ) in Beirut. All women included in our research were referred to the breast unit of the imaging department at Lebanese American University Medical Center Rizk Hospital (LAUMCRH). An informed consent was obtained from all the patients including explanation of the reason, modalities, risks and benefits of the biopsy procedure. All cancer biopsies pathological characteristics are detailed in Supplementary Table S1. One fragment of the breast biopsies for BiRADS cat 4 and 5 lesions under stereotactic guidance by VABB 7-11G or ultrasound guidance by Core needle 14G was sent to the Cancer and Metabolism laboratory of Saint Joseph University for molecular assessments. All samples were collected blindly without knowing in advance if the patient is healthy/normal or having the pathology. Identification of the tissue’s type was possible only after anapathological results. Normal/non-cancerous biopsied lesions are considered in this study the normal group. We confirm that all methods were performed in accordance with the relevant guidelines and regulations.

### Biopsies handling

All 79 biopsies were received each in a 10 ml tube of Ham’s F12 nutrient medium with 10%FBS and 1% Penicillin–Streptomycin-Ampicillin (PSA). According to their size, biopsies were washed between 3 and 5 times with 5 ml PBS(1x)-5%PSA at 1500 rpm for 5 min, then cut into two fragments subjected to RNA extraction.

### Cell culture

All cells used in this study were bought for the American Type Culture Collection (ATCC). MCF-7 breast cancer cells and MDA-MB-231 triple negative breast cancer cells were cultured in Dulbecco’s Modified Eagle Medium media supplemented with 10% Fetal Bovine Serum and 1% penicillin/streptomycin at 37 °C in a humidified incubator with 5% CO2.

### RNA extraction and reverse transcriptase polymerase chain reaction (RT-PCR)

Total RNA was extracted using TRIzol reagent (Sigma) according to the manufacturer’s instructions. RNA concentration and quality were determined via A260/230 and A260/A280 nm absorbance with Nanodrop Spectrophotometer. 500 ng of total RNA was subjected to reverse transcriptase (RT) reaction using iScript First Strand cDNA Synthesis kit (BIO-RAD) with random hexamer primers according to the manufacturer’s instructions (as previously described^50^).

### Quantitative reverse transcriptase polymerase chain reaction (qRT-PCR)

Briefly, the resulting double strand cDNA is subsequently analyzed by quantitative real-time PCR using the Rotor Gene Q technology and the QuantiFast SYBR Green PCR Kit (Qiagen) according to the manufacturer’s instructions. H19 levels were normalized to the expression of glyceraldehyde-3-phosphate dehydrogenase *(*GAPDH*)* serving as the internal control gene. Primer sequences for H19 are Forward: 5′TGCTGCACTTTACAACCACTG3′ and Reverse: 5′ATGGTGTCTTTGATGTTGGGC3′ and GAPDH Forward: 5′CACCCATGGCAAATTCCATGGC3′ and Reverse: 5′GCATTGCTGATGATCTTGAGGCT3′ (as described^50^).

### Statistical analysis

Statistical analysis was conducted using GraphPad Prism 6.01 software (as previously described^50^). The difference between groups was analyzed using unpaired or paired Student’s t-test when there were only two groups or assessed by one-way ANOVA followed by the Tukey’s multiple comparison tests when there were more than two groups. All tests carried out were two-tailed. Differences were considered as significant when p < 0.05.

### Ethics approval and consent to participate

The study was approved by the IRB of the Lebanese American University (LAU) and the ethical committee of Saint-Joseph University (USJ) in Beirut. Patients’ informed consent process is applied in the Lebanese American University Medical Center Rizk Hospital (LAUMCRH).

## Supplementary Information

Supplementary Table.
