# Supplementary material for: The long non coding RNA H19 as a biomarker for breast cancer diagnosis in Lebanese women
Source: Sci Rep. 2020 Dec 17;10:22228. doi: 10.1038/s41598-020-79285-z (PMC7747713; doi:10.1038/s41598-020-79285-z)
Supplement: Supplementary file 1 — Supplementary Table. [file 41598_2020_79285_MOESM1_ESM.pdf]

The long non coding RNA H19 as a biomarker for breast cancer diagnosis in Lebanese women

Tamina ELIAS-RIZK[1,3 §], Joelle EL HAJJ[2,3 §], Evelyne SEGAL-BENDIRDJIAN[4,5,6], George HILAL[3*]

[1] School of Medicine Lebanese American University, Beirut, Lebanon

[2] Natural Sciences Department, Lebanese American University, Beirut, Lebanon

[3] Cancer and Metabolism Laboratory, Faculty of Medicine, Saint-Joseph University, Beirut, Lebanon

[4] Team: Cellular Homeostasis, Cancer, and Therapies, INSERM UMR-S 1124, Université de Paris, France.

[5] Université de Paris, Paris Sorbonne Cité, France.

[6] BioMedTech Facilities, CNRS UMS2009/INSERM US36, Université de Paris, France.

[§] The authors contributed equally in the study

Tamina ELIAS-RIZK, email: tamina.rizk@lau.edu.lb

Joelle EL HAJJ, email: joelle.elhajj@lau.edu.lb

Evelyne SEGAL-BENDIRDJIAN, email: evelyne.segal-bendirdjian@inserm.fr

*Corresponding author: George HILAL, Cancer and Metabolism Laboratory, Faculty of Medicine, Saint-Joseph University, Mar Mikhaël BP 17-5208 1104, Beirut, Lebanon

Tel: 961 1 42 10 00; Email: george.hilal@usj.edu.lb

Supplementary table S1

Specimen number	BiRADS cat	Anapath	IHC	HER 2/Neu	Ki65	Agressive/poorly differenciated	Hormonal expression	Her 2
1	5	cancer	ER - PR-	1+	>80%	no	no	yes
2	5	cancer	N/A	N/A	N/A	N/A	N/A	N/A
3	4	cancer	ER++ PR++	0	14%	yes	yes	no
4	4 +micro	cancer	ER++ PR++	0	14%	yes	yes	no
5	4	cancer	ER++ PR++	0	NA	N/A	yes	no
6	5	cancer	ER++ PR+	2+	N/A	N/A	yes	2+
7	5	cancer	ER++PR-	0	low <5%	yes	yes	no
8	5	cancer	ER+ PR +	0	> 35%	no	yes	no
9	5	cancer	ER+ PR +	0	> 35%	no	yes	no
10	4	cancer	ER- PR -	0	20%	yes	no	no
11	5	cancer	ER++ PR++	0	< 5%	no	yes	no
12	5	cancer	ER+ PR +	0	N/A	N/A	yes	no
13	4	cancer	ER++ PR+	0	>60%	no	yes	no
14	4	cancer	ER- PR-	2+	20%	yes	no	yes
15	5	cancer	ER++ PR++	0	<5%	yes	yes	no
16	5	cancer	N/A	N/A	N/A	N/A	N/A	N/A
17	5	cancer	N/A	N/A	N/A	N/A	N/A	N/A
18	4	cancer	N/A	N/A	poor	yes	N/A	N/A
19	5	cancer	ER- PR -	0	> 40%	no	no	no
20	5	cancer	ER++ PR++	0	15%	yes	yes	no
21	5	cancer	ER++ PR++	0	10-15%	yes	yes	no
22	5	cancer	ER- PR-	0	> 40%	no	no	no

23	5	cancer	ER++PR++	0	< 10%	yes	yes	no
24	4	cancer	ER++PR++	0	< 10%	yes	yes	no
25	5	cancer	ER++PR++	0	2-3%	yes	yes	no
26	5	cancer	N/A	N/A	N/A	N/A	N/A	N/A
27	4	cancer	ER+ PR+	equivocal	10%	yes	yes	N/A
28	5	cancer	ER++ PR++	0	low index	yes	yes	no
29	5	cancer	ER++ PR-	1+	50-60%	no	yes	yes
30	5	cancer	ER++ PR++	0	20%	yes	yes	no
31	5	cancer	ER++ PR-	0	>30%	no	yes	no
32	4	cancer	ER++ PR-	0	<1%	yes	yes	no
33	4	cancer	N/A	N/A	N/A	N/A	N/A	N/A
34	5	cancer	ER++ PR+	0	5-10%	yes	yes	no
35	5	cancer	ER++ PR+	0	> 14%	yes	yes	no
36	4	cancer	ER++ PR+	0	<14%	yes	yes	no

Table 1: Detailed BIRADs category, hormonal expression, Ki index and HER2 expression of cancer biopsies.
